# Co-design of a voice-based app to monitor long COVID symptoms with its end-users: A mixed-method study

**DOI:** 10.1177/20552076241272671

**Published:** 2024-09-09

**Authors:** Aurélie Fischer, Gloria Aguayo, India Pinker, Pauline Oustric, Tom Lachaise, Paul Wilmes, Jérôme Larché, Charles Benoy, Guy Fagherazzi

**Affiliations:** 1 Deep Digital Phenotyping Research Unit, Department of Precision Health, 58942 Luxembourg Institute of Health, Strassen, Luxembourg; 2 Ecole doctorale BIOSE, 137665 Université de Lorraine, Nancy, France; 3 ACADI, Department of Precision Health, 58942 Luxembourg Institute of Health, Strassen, Luxembourg; 4 Association #ApresJ20 Covid Long France, Lucé, France; 5 549046 Luxembourg Centre for Systems Biomedicine (LCSB), University of Luxembourg, Esch-sur-Alzette, Luxembourg; 6 Department of Life Sciences and Medicine, 561906 Faculty of Science, Technology and Medicine, University of Luxembourg, Esch-sur-Alzette, Luxembourg; 7 Long Covid Center, Clinique du Parc, Castelnau-le-Lez, France; 8 82200 Centre Hospitalier Neuro-Psychiatrique Luxembourg (CHNP), Ettelbruck, Luxembourg; 9 University Psychiatric Clinics (UPK), 27209 University of Basel, Basel, Switzerland

**Keywords:** Long COVID, digital health app, mixed methods, vocal biomarkers, remote symptom monitoring

## Abstract

**Background:**

People living with Long COVID (PWLC), which is still a poorly understood disease, often face major issues accessing proper care and frequently feel abandoned by the healthcare system. PWLC frequently report impaired quality of life because of the medical burden, the variability and intensity of symptoms, and insecurity toward the future. These particular needs justify the development of innovative, minimally disruptive solutions to facilitate the monitoring of this complex and fluctuating disease. Voice-based interactions and vocal biomarkers are promising digital approaches for such health monitoring.

**Methods:**

Based on a mixed-method approach, this study describes the entire co-design process of Long COVID Companion, a voice-based digital health app to monitor Long COVID symptoms. Potential end-users of the app, both PWLC and healthcare professionals (HCP) were involved to (1) understand the unmet needs and expectations related to Long COVID care and management, (2) to assess the barriers and facilitators regarding a health monitoring app, (3) to define the app characteristics, including future potential use of vocal biomarkers and (4) to develop a first version of the app.

**Results:**

This study revealed high needs and expectations regarding a digital health app to monitor Long COVID symptoms and the readiness to use vocal biomarkers from end-users. The main expectations included improved care and daily life, and major concerns were linked to accessibility and data privacy. Long COVID Companion was developed as a web application and is composed of a health monitoring component that allows auto-evaluation of symptoms, global health, and scoring relevant symptoms and quality of life using standardized questionnaires.

**Conclusions:**

The Long COVID Companion app will address a major gap and provide day-to-day support for PWLC. However, further studies will be needed following its release, to evaluate its acceptability, usability and effectiveness.

## Introduction

Three years after the start of the COVID-19 pandemic, it has been estimated that a mean of 10–20% of COVID-19 patients will develop Long COVID, which represents at least 65 million people worldwide.^1–3^ The WHO definition of Long COVID states: “Post-COVID-19 condition occurs in individuals with a history of probable or confirmed SARS-CoV-2 infection, usually 3 months from the onset of COVID-19 with symptoms that last for at least 2 months and cannot be explained by an alternative diagnosis.”^4^ PWLC present complaints such as tachycardia, extreme fatigue, dyspnea, and inability to perform daily physical tasks.^5^ More than 200 symptoms have been associated with Long COVID, and multiple organs are affected.^3^ Our previous work showed that 59% of COVID-19-infected people from the Predi-COVID cohort study reported one or more persisting symptoms after a year. The number of persisting symptoms increased with the initial disease severity, and the quality of life of those participants was notably impacted by sleep disorders (54%) and compromised respiratory function (12.9%). Nonetheless, individuals with an initially asymptomatic or mild form of COVID-19 infection could also be affected.^6^

At an individual level, the impact on the daily life of people affected by Long COVID is high, with many people who do not return to the same physical or professional activity level. Impact on society, in general, is also considerable with increased costs related to sick leaves, handicapped workers, and long-term condition recognition.^7^ In particular, 62.2% of people with Long COVID stopped working, and only 32.5% resumed full-time professional activities.^8^ People experiencing long-term consequences of COVID-19 frequently encounter problems in obtaining a Long COVID diagnosis and accessing specialized health services.^9–11^ Despite the increase in knowledge on Long COVID and an increasing number of medications under clinical investigation, there is still no evidence-based treatment.^12^

Long COVID disease management consists mainly of the integration of different strategies combining treatments for some specific symptoms, such as neurocognitive, physical, taste and smell rehabilitation, and dietary or activity recommendations. Pacing is the main activity management recommendation and is an approach to balancing activities with rest to avoid exacerbation of symptoms.^13^ In the absence of validated treatment, people experiencing persisting COVID-19-related symptoms are, therefore, in need of a tool to monitor the progression of their symptoms and to help them with general disease management. In addition, a panel of experts from the National Institute for Health and Care Excellence (NICE) recommended the development of telemonitoring and encouraged self-management of acute and Long COVID symptoms in a tailored and accessible way for each patient.^14^ Upon the availability of new treatment, this tool could also be used as a proxy to measure the improvement of global health and specific symptoms.

Long COVID care, as for other chronic diseases, should align with the concept of minimally disruptive medicine, aiming for a reduced burden on patients’ lives while maximizing health outcomes. PWLC frequently have several healthcare professionals (HCPs) in charge of the different aspects of their care, with many appointments and travels to manage. They are also regularly asked to complete long standardized questionnaires or scales to evaluate their symptoms, which generates an avoidable burden on their lives if care is not coordinated. The development of innovative methods to integrate multiple patient report outcomes in a portable, versatile way, to reduce travels for medical care, and overall for remote health monitoring is therefore of the highest importance.

Voice is a promising candidate increasingly used in mobile health (mHealth) interactions in chronic diseases.^15–17^ Even if there is no clear evidence so far, we believe that it may be easier and more inclusive for patients with chronic diseases than completing questionnaires for example. Voice is an easy and cheap medium to collect and can be easily integrated into a device like a smartphone, now widely used by people of different ages and education levels. Voice dictation has also entered into the habits with the development of voice messages and home assistants like Google Home or Alexa.^18^ It can also be used for vocal biomarker assessment as new clinical endpoints for relevant symptoms. However, no validated vocal biomarkers are currently available as a standard of care and there is a need to clinically validate vocal biomarker candidates to bring them into clinical and real-life practice. Vocal biomarkers have already been described in different therapeutic areas like Parkinson's disease,^19^ mental health, cardiovascular diseases or diabetes.^18^ Some of them have been successfully integrated in smartphone apps, currently available on the app stores, like the Real Time Voice Analyser app to monitor respiratory wellness,^20^ or the Sonde Mental Health app, based on a vocal biomarker of mental fitness.^21,22^

In Luxembourg, participants from the Predi-COVID hybrid prospective cohort study were invited to perform voice recordings simultaneously as the filled-in online questionnaires regarding their symptoms and health status. To date, almost 6000 voice recordings from more than 500 COVID-19 patients have already been collected in the Predi-COVID study.^23^ These voice recordings have been analyzed, and vocal biomarker candidates have already been identified with performances above 80% to detect fatigue, loss of taste and smell, and symptomatic status in COVID-19-infected people.^24–26^ However, further progress is required before these vocal biomarkers can be used in clinical practice. They must be validated with new investigations and there is still a need to develop vocal biomarkers for other symptoms such as respiratory problems.

Many symptom monitoring apps exist for different use-cases including those tailored for patients undergoing chemotherapy treatments, people with mental health conditions, or those experiencing chronic pain. They represent an additional tool for managing symptoms that could be integrated into the care of patients, as a regular use may alleviate the symptom burden and improve quality of life by limiting travels for medical appointments. In the case of Long COVID, people with persisting symptoms often experience difficulties communicating with HCPs and sometimes do not feel believed. Therefore, a monitoring app could also facilitate the communication between them.

Regarding the existing apps that could meet the needs of people with Long COVID, some already exist, such as “Visible”^27^ or “Living With.”^28^ However, these apps are only available in the US and in the UK, respectively. Furthermore, the “Living with” app is available only by invitation. This limits their availability for PWLC in Europe. Some other apps designed for other chronic conditions, like myalgic encephalomyelitis/chronic fatigue syndrome (ME/CFS) Pacing app, are also used by some people with Long COVID to manage their daily energy levels. However, these apps do not fully meet the specific needs of people with Long COVID, and are only available in specific countries, in English, and some of them only on the iOS operating system, which restricts their potential users. Moreover, to the best of our knowledge, none of the above-mentioned apps were the subject of a scientific peer-reviewed publication describing their development and none of them are embarking vocal biomarkers or voice in general.

Co-designing mHealth solutions with end-users and HCPs is crucial to ensure adherence and clinical relevance. Various study designs, based on qualitative methods alone or on mixed-methods, blending qualitative and quantitative methods, have already been used to assess the user's needs and develop digital health solutions in different therapeutic areas.^29–32^

The overall objective of our study was to co-design a voiced-based digital health app available to the greatest number, with people affected by Long COVID and HCPs in charge of Long COVID patients.

To achieve this objective the study consisted of four sub-objectives: (1) to understand the unmet needs and expectations related to Long COVID care and management, (2) to assess the barriers and facilitators regarding a health monitoring app, (3) to define the app characteristics, including future potential use of vocal biomarkers, and (4) to develop a first version of the app.

## Methods

### Patient and public involvement

Integrating patient and public involvement approaches in research and e-health co-design processes is key to ensure maximal adoption and to make sure the solution responds to patients’ needs and priorities.^33,34^

The collaboration with patient partners of the association #Apresj20 Covid Long France^35^ was established in previous research,^6,36^ facilitating their involvement from the beginning of the study. By raising their need for a monitoring tool, they were at the origin of the app development. This app is therefore made by and for people with Long Covid.

More specifically, three Long COVID patients and two HCPs were involved as co-researchers in the study design and at all research stages by reviewing the protocol, the interview guide, the survey questions, and the informed consent forms, to ensure that the objectives were pertinent and that the study participation induced no exceeding burden. They were also involved in the recruitment by disseminating the information on the study via social media and internal communication within the #Apresj20 association members. Finally, they will be involved in the dissemination of study results through the #Apresj20 association communication channels and by directly informing their patients (for HCPs).

### Study participants

Study participants were adult people (women and men) experiencing Long COVID (diagnosed or not, to ensure a good coverage of all PWLC) and HCPs in charge of Long COVID patients. All participants were native French speakers.

To recruit participants, electronic and paper flyers presenting the study were disseminated via social media, Long COVID dedicated consultations, and Long COVID patient associations in Luxembourg and France. Participants from the Predi-COVID cohort study^23^ who declared persisting symptoms one year after the acute infection were also invited to participate. The detailed recruitment and enrollment process in the study is detailed in the published study protocol.^37^

### Study design

The UpcomingVoice study is a cross-sectional study based on a mixed-method sequential exploratory design. Quantitative and qualitative data were collected concurrently and analyzed separately. It consisted of two successive phases:

1. A quantitative phase based on a web-based anonymous survey (descriptive approach).
2. A qualitative phase based on semi-structured individual interviews (inductive pragmatic approach) and a focus group.

**Figure 1.**
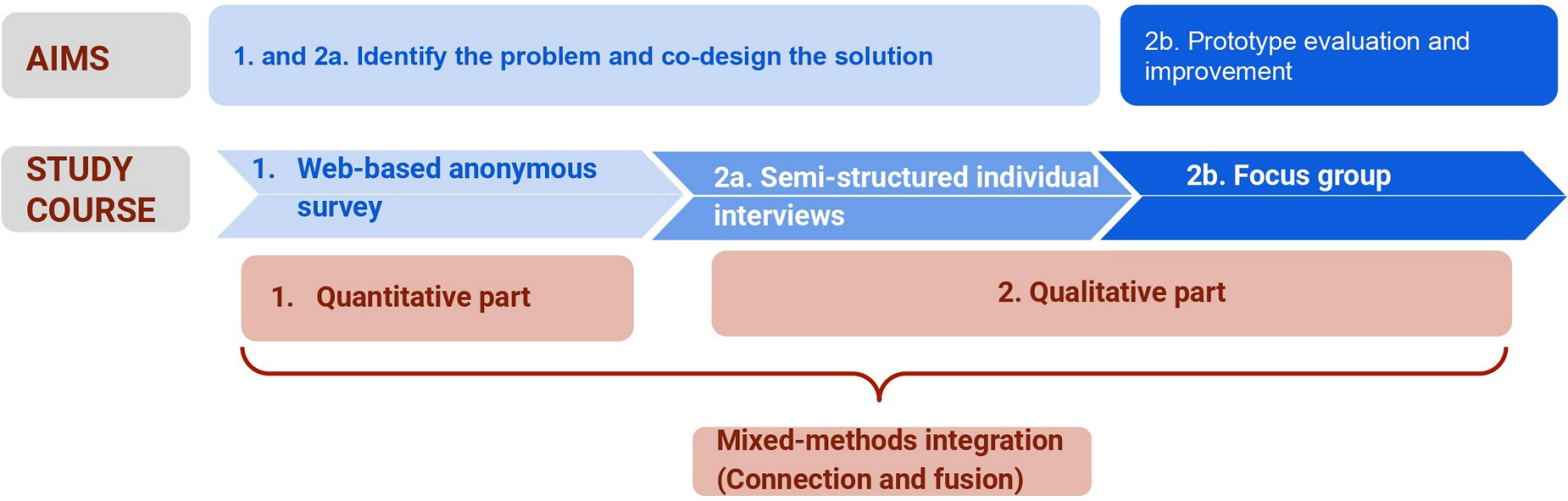
Study design.

The study design and aims are summarized in Figure 1. The study was conducted in French, as participants were coming from French-speaking countries.

#### Quantitative phase

The quantitative part of the study consisted of an anonymous web-based survey addressed to both PWLC and HCPs. The survey was conducted between October 2022 and April 2023.

This part aimed to define the main aspects of daily life most impacted by Long COVID, the needs of PWLC, to assess acceptability, and expectancies toward the use of voice in a digital health app, and to define the general outlines of the smartphone app.

We used ad-hoc questions covering topics like Long COVID symptoms and impacts, fears and expectancies toward a smartphone app to monitor symptoms alongside the use of voice in such an app, and the five main aspects of the User Version of the Mobile Application Rating Scale (uMARS):^38^ Engagement, Functionality, Aesthetics, Information, and Subjective items (“Would you recommend,” “Would you be interested in,” etc.). We chose to base our questionnaire on these five uMARS themes because uMARS is the most used questionnaire for evaluating the quality of mHealth apps, so it was important to take it into account as soon as possible in the app development.^39^

The survey questions were adapted depending on the participants’ type (PWLC or HCP). Some questions or answer modalities were common, but others were specific to one participant's type. The detailed PWLC and HCP questionnaires were provided in the study protocol.^37^ Survey completion took a maximum of 20 min to ensure a high acceptability rate, particularly in people with Long COVID who frequently experience fatigue and trouble concentrating. Survey data were collected and managed using REDCap electronic data capture tools hosted at the Luxembourg Institute of Health.^40^

#### Qualitative phase

The qualitative phase of the study included semi-structured individual interviews (2a) and a focus group (2b).

2a. The semi-structured individual interviews aimed to go deeper into the definition of PWLCs’ problems, needs, fears and expectancies, and the characteristics of the smartphone app. To ensure good interview quality, the following measures were taken. First, the interviewers introduced themselves and reminded the overall study aims and what were the specific aims of individual interviews. Second, participants were reminded that there was no good or false answer and that they could refuse to answer a question they did not want to answer. Finally, PWLC participants were informed to ask at any time to take a break or to stop the interview in case they felt too tired, to limit the risk of post-exertional malaise. Survey results on related questions were not communicated to participants to not influence their answers and opinions. The interview guide was developed to assess the study objectives in more depth than the survey. Moreover, an intermediary analysis of survey results was used to identify the most important concerns and to discuss them at the beginning of the interviews to ensure sufficient time to address them correctly. Finally, interviews were organized in the following sections: Understanding Long COVID impact on life, identifying expectations, fears, barriers and facilitators regarding a smartphone app and the use of voice, and defining the app characteristics. Individual interviews were conducted between March and April 2023. Individual interviews had a mean duration of 44 min (range: 24–68 min).

2b. Focus group: A first version of the app has been developed based on specifications defined with survey and individual interview results. It was presented to a panel of PWLC and HCPs during a focus group of 90 min organized in January 2024. The focus group was structured as follows: After a short reminder of study and focus aims, the moderator made a demo of the app, then each main functionality was individually discussed. Finally, feedback and improvement suggestions were collected during a semi-guided discussion.

The individual interview and the focus group guides are provided in Supplementary file 1. Each main topic was introduced by a leading question. Ad-hoc, open-ended questions were used to encourage the discussion and the emergence of potential new topics. Stimuli questions were prepared and used only in case participants were silent or did not get the topic clearly.

Both parts were conducted by an experienced researcher in epidemiology and infectious diseases with additional training in mixed methods, who had no prior relationship with the participants. Individual interviews and the focus group were done using a web-based teleconferencing system, and where content was audio recorded, transcribed into verbatims and anonymized. Pilot sessions of both the interviews and the focus group were organized to limit the risk of technical issues and to ensure respect for the duration of time. The participants could participate in the survey, individual interview and focus group, but their data were not linked.

#### Population size

*Quantitative part:* Due to the exploratory nature of the study, a formal size calculation for the survey was not possible. The quantitative data collected through the survey will be solely utilized for descriptive analysis and to assess the significance of various topics for the participants.

*Qualitative part:* Qualitative research does not explicitly aim to secure a representative sample of individuals, but aims to capture diversity. No gold standard to estimate the correct sample size presently exists.^41^ Individual interviews were continued until data saturation was achieved, which can be defined by the point at which no new themes emerge.^42,43^ A recent systematic review showed that data saturation was achieved with a mean of 9–17 interviews, but with high disparities between studies.^43^ We estimated that including at least 15 PWLC and five HCP for the individual interviews would allow us to achieve data saturation. We also actively monitored the inclusions for the individual interviews to ensure a balanced representation across various age groups and gender categories, thereby enhancing the diversity within our study population. For the focus group, it was estimated that the ideal sample size was between six and 10 participants, particularly when involving persons highly informed on the topic.^44^ Participants of the individual interviews who expressed their interest in participating in the focus group were invited to this.

### Data analysis

#### Survey

Quantitative data resulting from the web-based survey were analyzed using descriptive statistics. We described the normally distributed continuous variables as mean (min–max), while the categorical variables as numbers (percentage). We used the Student’s *t*-test, the one-way analysis of variance (ANOVA) to determine the differences of distribution for continuous variables and Fisher's exact tests for categorical variables. We performed all the analysis using the R software version 4.3.1.^45^

#### Semi-structured individual interviews

The Maxqda software was used for transcription of interviews recordings and for the coding and analysis of transcriptions. The quotes presented in the present study were translated from French to English and are cited using an arbitrary participant number, for example, PWLC1 or HCP1.

We applied inductive reflexive thematic analysis to find patterns in the data and define the main topics of interest among the three aspects discussed (understanding Long COVID impact, identifying the needs, and defining the app characteristics). Two experienced researchers were involved in the coding process (AF and GA). The first step was the familiarization with the whole data set by the two researchers. The second step was the generation of initial codes separately by AF and GA. Codes were then compared and refined to group them by themes. An iterative process of discussions between the two researchers allowed coding structure consolidation.

After 16 interviews with PWLC and five with HCPs being realized, transcribed and analyzed, the research team (AF and GA) determined that data saturation was achieved as no new theme or subtheme reached out anymore.^42^

#### Focus group

The Maxqda software was used for the transcription of the focus group audio recording and for analysis of the transcript. The feedback obtained was summarized and grouped by the five themes from the uMARS: engagement, functionality, information, aesthetic, and subjective items.

#### Mixed-methods integration

Data integration was done through two mechanisms:^46^

*Connection:* Survey results were used to elaborate the individual interview guide so that the most important aspects could be discussed in priority during the interviews.

*Fusion:* Data from survey and individual interviews were grouped by themes and compared to determine the areas of convergence, divergence, and expansion.^47^ ‘Convergence’ describes a positive alignment between the survey respondents and the interview participants. ‘Divergence’ describes a disagreement between qualitative and quantitative findings. Expansion indicates that qualitative and quantitative data addressed the same concept but in different and complementary ways.

### Ethics

The study was approved by the National Research Ethics Committee of Luxembourg (study number 202208/04) in August 2022. No explicit informed consent was required for the participation in the quantitative part, based on an anonymous survey. An explicit informed consent was electronically signed by all participants of the qualitative phase before participating in the individual interview.

## Results

### Study population

The survey was completed by 201 PWLC and 15 HCP. PWLC were mostly women (83.1%), with a mean age of 46.9 years (min 18–max 92), and were in majority of higher education (undergraduate or more). HCP were also mostly women (60%), mean age was 40.5 years (min 27–max 77) and 93.3% of them were of higher education. Fifteen PWLC and five HCP participated in the individual interviews and among them, six PWLC and three HCP also participated in the focus group.

The characteristics of the survey, interviews, and focus group participants are summarized in Table 1.

**Table 1.** Characteristics of participants.

		Survey		Interviews		Focus group	
Variable		HCP (*n* = 15)	PWLC (*n* = 201)	HCP (*n* = 5)	PWLC (*n* = 16)	HCP (*n* = 3)	PWLC (*n* = 6)
Females, *n* (%)		9 (60.0%)	167 (83.1%)	2 (40%)	10 (62.5%)	1 (33.3%)	2 (33.3%)
Age, mean (min–max)		40.5 (27–77)	46.9 (18–92)	42.0 (35–54)	51.4 (28–68)	41.3 (35–54)	49 (28–60)
Level of education, *n* (%)	Lower secondary education	—	27 (13.4%)	—	3 (18.7%)	—	—
	Upper secondary education	—	32 (15.9%)	—	2 (12.5%)	—	1 (16.7%)
	Bachelor's degree	6 (40%)	61 (30%)	1 (20%)	4 (25%)	1 (33.3%)	2 (33.3%)
	Master's degree or higher	9 (60%)	80(40%)	4 (80%)	7 (43.8%)	2 (66.7%)	3 (50%)

PWLC survey participants were mostly not hospitalized during their initial COVID-19 infection (80%). Initial symptoms were mild for 55% and severe for 37% of them. 79% experienced severe persisting symptoms and 91% already obtained a Long COVID diagnosis.

For all survey answers provided below there was no influence of age, gender, or level of education for PWLC or for HCP (data not shown, available upon request).

### Diagnose: identify the problem and need

The results of the survey and individual interviews were first grouped in categories: LC impact on life, interest and acceptability regarding a voice-based app, and considerations for a digital health app. For each category, we grouped results in theme and sub-themes.

#### Long COVID impact on life

For this category, we identified two main themes: daily life impact and disease management.

In relation to the theme “daily life impact,” 88% of the survey participants declared that they did not recover the same level of professional and leisure activity as before COVID-19 infection.

Five sub-themes from the “daily life impact theme” were identified from the individual interview transcriptions: feelings, financial issues, work issues, social life, and daily functioning. In relation to the psychological impact, both HCP and PWLC reported that Long COVID was a source of anxiety and stress. As mentioned by HCP 12, “It's a source of psychological difficulties for them, as they find themselves in a completely unexpected state.” Moreover, many PWLC underlined that they often feel alone or misunderstood, either by their close-relatives or by HCPs. “Honestly, I’m lost. I feel completely alone and powerless” (PWLC 57). Financial issues that were mentioned were financial precarity, due to an income reduction, or the cost of medical care. This was closely related to work issues, with half of the PWLC explaining that they had to stop, reduce, or adapt their professional lives. Some of them also felt they were at risk of losing their position because of committing many errors due to cognitive problems. The high impact on social life was also often reported by HCP and PWLC, with fewer leisure activities, fewer activities with family and friends, less pleasure in eating or going out to restaurants due to loss of taste or smell or to cognitive problems. Finally, simple tasks from daily functioning were impaired, as PWLC 62 explained: “On a day-to-day basis, I have to choose between taking a shower and taking out the rubbish.” Extreme fatigue was the center problem that induced difficulties in daily tasks.

Disease management was an additional important theme related to the impact of Long COVID on lives. 64% of survey participants with Long COVID already benefited from a specialized Long COVID consultation. However, 60% of the survey participants declared that access to care was difficult or very difficult. Three sub-themes could be identified from the individual interviews: Access to care, disease recognition, and rehabilitation. Some inequities in the access to care were reported by PWLC, with people having problems obtaining an appointment with the right specialist, with specialized Long COVID centers being overwhelmed and with people not even knowing that specialized Long COVID centers exist. Disease recognition was also reported as a problem, by PWLCs and HCPs, who mentioned that some HCP don’t know much about Long COVID and some of them even don’t believe their patients and the existence of Long COVID. “At least half the doctors I see don’t believe in my symptoms and tell me that it's all in my head” (PWLC 23). The recognition of Long COVID as a long-term disease was also a problem with administrative issues coming in addition to the lack of knowledge on Long COVID from some HCPs. The last theme of concern was rehabilitation. While some rehabilitation exercises and pacing seem to be helpful, some PWLC and some HCP underlined that rehabilitation may not be adapted “You can tell people to do sudoku or memory, but it won’t necessarily help them with their shopping list or their job.” (HCP 7) or even harmful with a risk of post-exertional malaise “I believed in exercise rehabilitation, and then I had a relapse.” (PWLC 43). Rehabilitation also induces many appointments which can be tiring and burdensome.

Interview results were in convergence and expanded upon the survey results, and provided more in-depth information on areas impacted by Long COVID in their lives. Survey and interview themes and verbatims are summarized in Supplementary Table 1.

**Figure 2.**
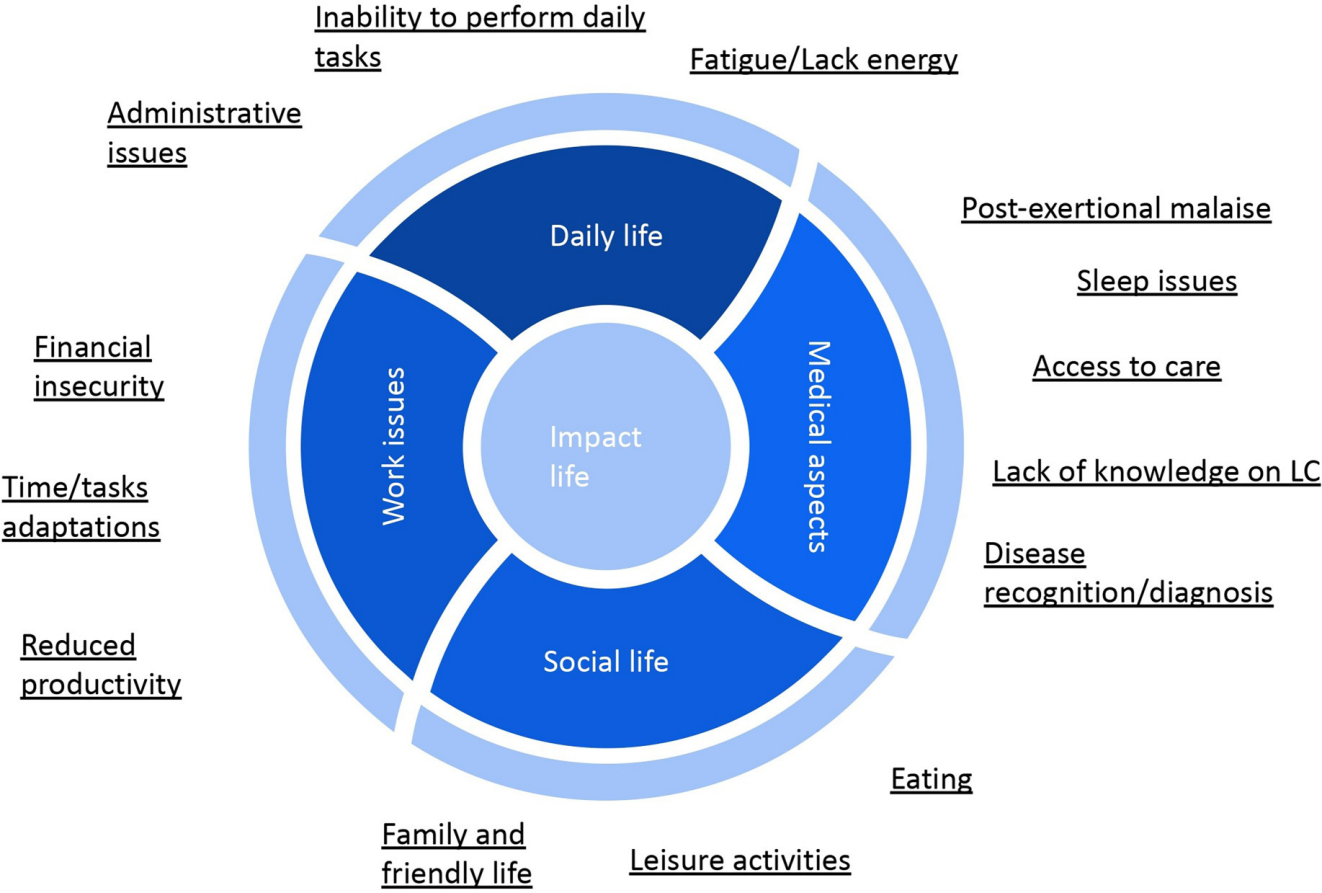
Conceptual framework of key aspects of long COVID impact on daily life.

Based on these results, a conceptual framework of key aspects of Long COVID impact on affected people has been elaborated based on these results (Figure 2).

#### Interest in and acceptability of a voice-based digital health app

People with Long COVID were 51% to think that a digital health app could be useful to manage their health in the long term, and 43% answered that they did not know.

However, 82% of PWLC declared that they would be interested or very interested in a digital health app based on vocal biomarkers to monitor symptoms.

HCPs were 82% to think that a voice-based monitoring app would be useful for people with Long COVID, regardless if they are already in dedicated Long COVID care or not, and 73% of them would recommend the use of this kind of app.

Finally, 70% of PWLC and 60% HCP agreed that the app should include both voice recordings and questionnaires. Complete survey answers regarding interest and acceptability of an app based on vocal biomarkers (VB) are provided in Supplementary Table 2.

During the interviews, it appeared that all HCPs and PWLC were favorable to the use of voice and vocal biomarkers. Among the advantages that were raised during interviews was the reduction of travels for medical visits: “Using voice is intuitively logical for patients, to limit travels” (HCP 66). The ease-of-use of voice compared to writing was also raised by several participants: “I like the idea of voice, because it could be a way of reducing screen fatigue, rather than writing.” (PWLC 16) “There's an aspect of simplicity and reproducibility that's quite easy for patients, who can do it whenever they want. They can do it at home and not just when they see the doctor.” (HCP 9) “I’d like to have voice monitoring, because I find it less tiring than having to write down all the symptoms.” (PWLC 14)

HCP 37 also positively regarded vocal biomarkers: “It's something that can be more powerful than standardized questionnaires.” However, it was highlighted that it is important to demonstrate that vocal biomarkers are accurate to gain trust from the users, as mentioned by PWLC 19: “What's important here is to gain trust and demonstrate that it can be useful and how it can be useful.”

Another advantage of vocal biomarkers would be the early detection of symptom worsening or improvement. For example, HCP 7 said: “If it can detect maybe a little bit in advance, maybe even a little bit before people realize and that would be really very useful for our patients,” meaning that patients could slow down their activities before the worsening becomes too much disabling. On the other hand, HCP 37 saw it differently and said: “If things get worse, the patient realizes it anyway. A biomarker can be useful precisely to show that things are getting better. That could be interesting and encouraging.”

Finally, an expectation raised from PWLC is that vocal biomarkers could be more objective than an auto-evaluation. “It's something measurable, something more objective, in quotation marks, than the personal assessment we can make of our symptoms.” (PWLC 19) “I just think it's more objective than giving a score to everything over time.” (PWLC 21)

#### Considerations regarding the digital health app

##### Expectations

The main expectations identified in the survey were: (1) a better symptom monitoring (PWLC 71.1%, and HCP 80%) and to obtain an objective measure of symptoms with vocal biomarkers (PWLC 56%, and HCP 53%, (2) the evaluation of a rehabilitation program (PWLC 48%, and HCP 73%), (3) to limit the medical visits (PWLC 31%, and HCP 47%, and (4) to obtain a Long COVID diagnosis (PWLC 43%, and HCP 33%). During the interviews, two main themes related to expectations were identified: care and daily life. The “Care” theme can be divided into four sub-themes: symptom monitoring and identification, improvement of communication between PWLCs and HCPs, improvement of care and access to care, and the limitation of medical visits. This confirmed survey results except for the Long COVID diagnosis, which did not emerge during the interviews as an expectation. Symptom monitoring was the central expectation. It also came out that identification of symptoms was an important aspect for some PWLC who find it difficult to describe their symptoms with the right words. Some PWLC mentioned that they already tried to monitor their symptoms using a paper diary but find it not practical. (“I have a diary to write down my symptoms. So, I write them down sometimes, but it's true that it's not super practical.” PWLC 44). This confirmed that a digital health app could be an added value for them. A HCP suggested that symptom monitoring and its visualization may even have a therapeutic interest (“Having a calendar of symptoms like this can be very therapeutic (…) to show the fluctuation of symptoms, to show and visualize the course of certain symptoms” HCP 66). Symptom monitoring over time was also expected to be more objective than the subjective perception that a person can have about his/her health status.

The “Daily life” theme comprised two sub-themes: psychological support and administrative support. PWLC participants were a majority to report high levels of stress and anxiety. Some of them said that they expect the digital health app to provide them psychological support in one way or another: “I don’t know where or how it can be set up, but here it is, psychological help.” (PWLC 55) “Measuring and sending back information in relation to what we have can be very interesting and reassuring too.” (PWLC 44). HCPs had a similar opinion, as they were 53% to think that this kind of app could be a companion tool for PWLCs and HCP 7 said during the interview that it could help them “To feel less alone, to feel understood, to not feel abandoned.” The “administrative support” theme included administrative information linked to the medical aspect, return to work and patient's association contacts.

##### Concerns

Survey results showed that the most frequently reported barriers by PWLCs were: a wrong result interpretation (67.7%), symptom intensity (37.3%), and data protection (36.3%). Among HCPs, the three most important barriers were: a wrong result interpretation (66.7%), data protection (53.3%), and age (26.7%). Age was reported as a barrier by only 24% of PWLC and symptom intensity by only 6.7% HCP.

The interviews confirmed the survey findings, with two main themes. The first theme was “accessibility” encompassing illiteracy, vision or language problems, and an excessive volume of content that could complexify too much the app use. The second theme was “user confidence” and covered confidentiality issues and the trust in a new technology like vocal biomarkers. The confidentiality issues were either general to the digital health app with participants expressing some concerns with sharing personal and health data (“I’m always a bit suspicious, I prefer to keep the collection of personal and health data to the strict minimum. PWLC62 or “We’re perhaps a little afraid, a little suspicious, so I think it's really a question of developing trust, of saying that this is really for the user, … and also to show that RGPD is respected.” PWLC 19”) or specific to the use of voice that could be a barrier in the app use settings (“Everyone around you hears what you’re saying, and I think that can be a bit of a barrier for some people, who will wait until they’re alone, until they’re well isolated, to use the app, because they have to use their voice.” HCP 7). Finally, as vocal biomarkers are a recent technology it was raised as a barrier by some participants who need to be convinced that they can trust it, as mentioned by PWLC 16 “Since I don’t know anything about it, I’m wondering about its reliability.” and PWLC51 “It remains to be seen whether it really works.”

##### Facilitators

The main facilitators identified in the survey were the app ease-of-use (PWLC 72%; HCP 73%), the vocal biomarkers results visualization (PWLC 61%; HCP 47%), and reliability (PWLC 54%; HCP 80%).

During the interviews, two themes emerged (user engagement and app content). Facilitators linked to user engagement were adaptability/personalization (“It has to be general, but I’d like to adapt it to my own needs.” PWLC 57; “The more customized, the better” PWLC 19; “Adaptability to each patient is the key word in care. For an application, I think it could be good” HCP 7), an easy-to-use conception (“I’d like something very simple.” PWLC 16; “In terms of access and interactivity, it has to be simple.” PWLC 44), integration of voice dictation (“I do almost all my stuff orally because I’m tired of writing,” PWLC 14; “it might help some people who are less at ease with the written word.” PWLC 4), and result visualization from vocal biomarkers but also generally speaking about symptom evolution (“It could be something really appreciated by patients to have regular feedback with nice graphics.” HCP 66; “when you see a curve that goes up and down, it's worth a thousand words.” HCP 9).

Expectations, facilitators, and barriers, with verbatim quotes are summarized in Supplementary Table 3.

**Figure 3.**
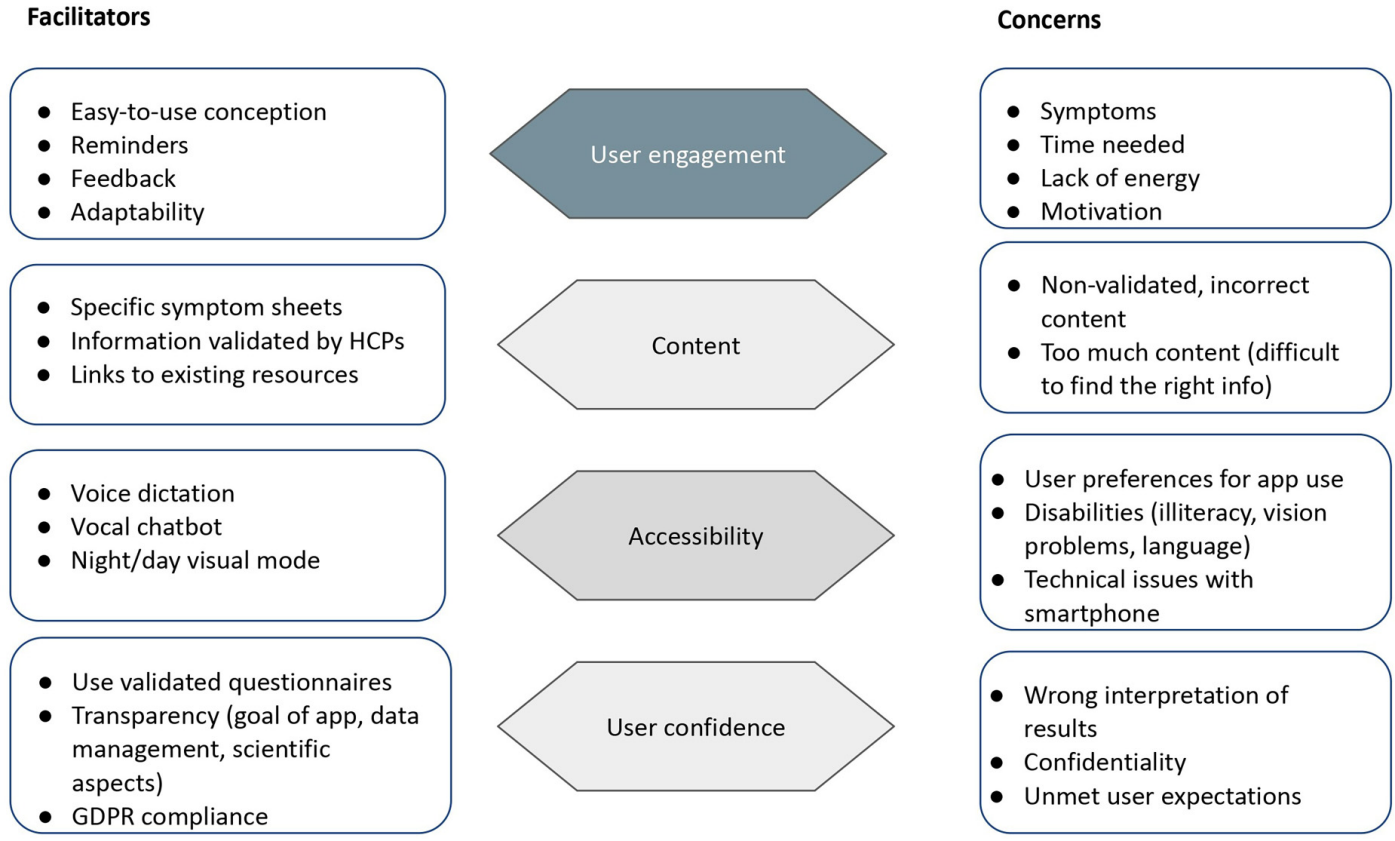
Concerns and facilitators regarding a digital health app.

To summarize these results, concerns and facilitators were grouped into four main themes (user engagement, app content, accessibility, and user confidence) and presented in Figure 3.

### Define solution: App characteristics

#### App features

Survey results and interview verbatim quotes were grouped according to the five themes of the uMARS: engagement, functionality, aesthetics, information, and generalities.

Regarding the engagement theme, interviews revealed that personalization options were not essential and that interaction with other app users, namely other PWLC, was rather seen negatively, with the need of a moderator, the presence of potentially toxic people, and did not appear to be a real need for PWLC.

**Figure 4.**
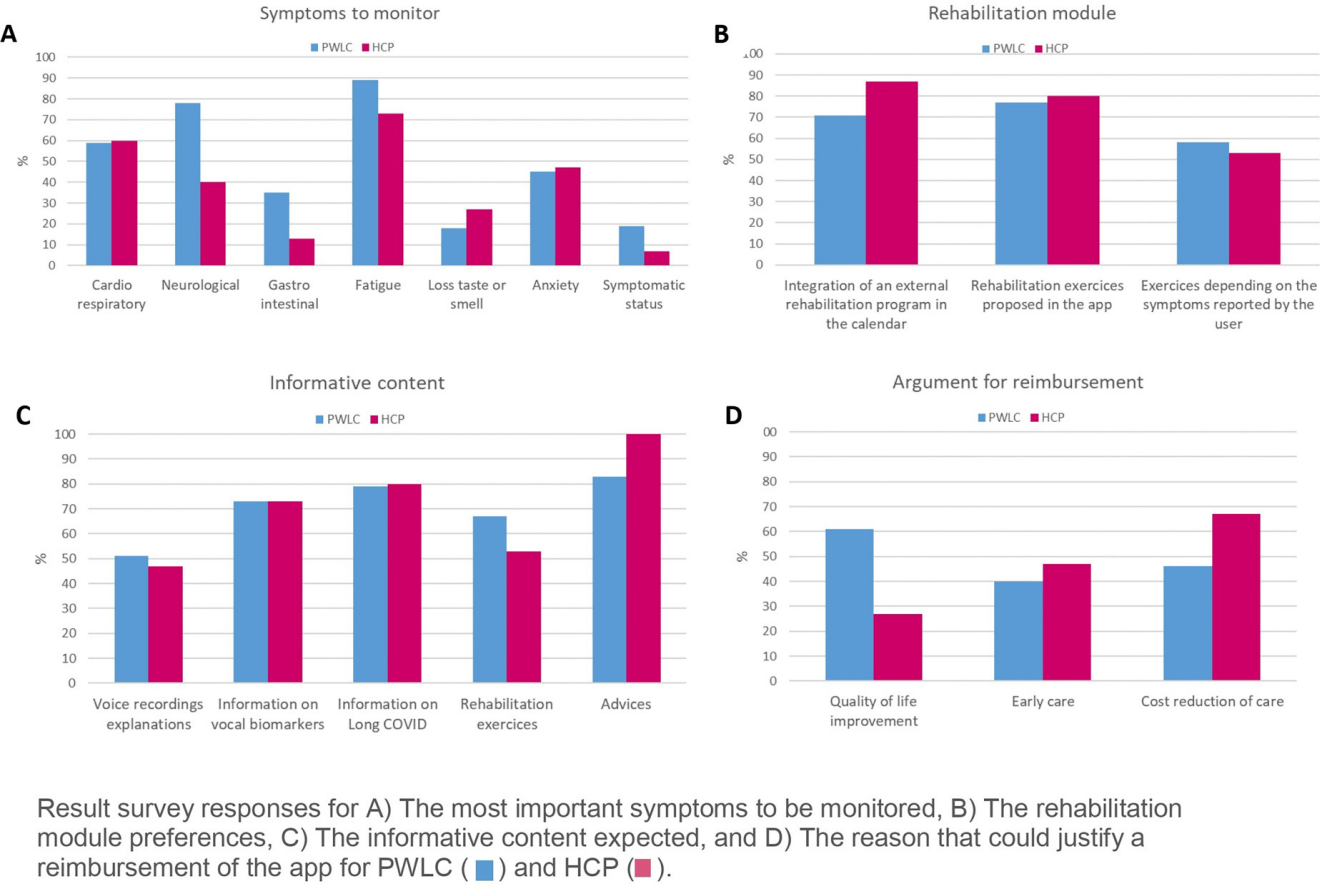
Survey findings.

App functionalities were extensively discussed with all interview participants. Survey results and individual interview verbatims related to app characteristics are provided in Supplementary Table 2 and some detailed survey answers are summarized in Figure 4.

The symptom monitoring was essential and the main symptoms to monitor were cardiorespiratory and neurological symptoms, fatigue, and anxiety. It came out that the app should allow the monitoring of several symptoms and should be based on vocal biomarkers and validated questionnaires. Participants also reported that it would be interesting to have a list of symptoms and to be able to indicate the perceived symptom intensity.

The alert system component which seemed to be important for 63% of PWLC and 73% of HCP survey respondents was not consistent among the interview participants. Several participants mentioned that this could be interesting if interactions with HCP were implemented but otherwise, if the user received the alert it could be a source of additional anxiety. Participants suggested an alternative option would be to simply give advice to rest or to consult a doctor.

The integration of rehabilitation exercises in the app was expected by 77% of PWLC and 80% HCP survey respondents. However, when discussing this topic during interviews, it was less clear. Indeed, some participants thought that it could be good to propose breathing, cognitive, or even physical reinforcement exercises (“Suggest, for example, different types of activities, relaxation strategies.” PWLC 19; “perhaps neurocognitive re-education exercises could be added” HCP9), but several others mentioned that there could be a risk of post-exertional malaise, and that in cases where exercises were proposed they should be accompanied through an extensive amount of detailed explanations and pacing should be explained (“Not pushing yourself too hard, or restricting yourself too much. And that's not easy. So, with the right explanations about post-exertion discomfort and all that, yes” PWLC 72; “(NB: when discussing rehabilitation exercises): It contributed to me having another Post-Effort Malaise crash, you see? And a big crash.” PWLC 44).

Among PWLC survey respondents, 76% answered that they would have the option to share their data and results with their family or with HCP. 34% expected to be able to send emails to HCP through the app. Interview participants, either PWLC or HCP, were not that much convinced about the necessity and feasibility of implementing the possibility of a direct interaction between PWLC and HCP through the app. The main need seemed to have a synthetic and graphical view of app data and results to serve as a discussion basis during consultations. Therefore, the option to generate a downloadable PDF report emerged as an efficient and easy alternative.

During the interviews, an additional need emerged, namely the integration of a medical and daily life diary, so that PWLC could record important information like treatments, medical assessments, medical events, physical and cognitive activity or level of stress.

Regarding the informative app content, survey results showed that informative content was expected by 87% of PWLC and 87% of HCP. Advice was expected to be present in the app by 83% of PWLC and 100% HCP. During interviews, these results were confirmed. However, it also came out that the app should not contain too much information and that information and advice should be validated and up to date. Some participants mentioned that internet sites with a lot of information already exist; “You could list a number of sites where people can connect, I don’t think the application should become a Long Covid Encyclopedia.” (HCP 9). Personalized advice would be interesting as mentioned by PWLC 4 “Advice on matters that concern me” but many participants agreed that advice on pacing, physical activity, sleep and diet would be interesting for the majority of users.

Finally, 83% of PWLC and 73% of HCP survey respondents thought that the app should be reimbursed, but this point has not been discussed during interviews and did not emerge spontaneously. The main reasons in favor of the reimbursement were quality of life improvement for 61% of PWLC and the reduction of care costs for HCP.

**Figure 5.**
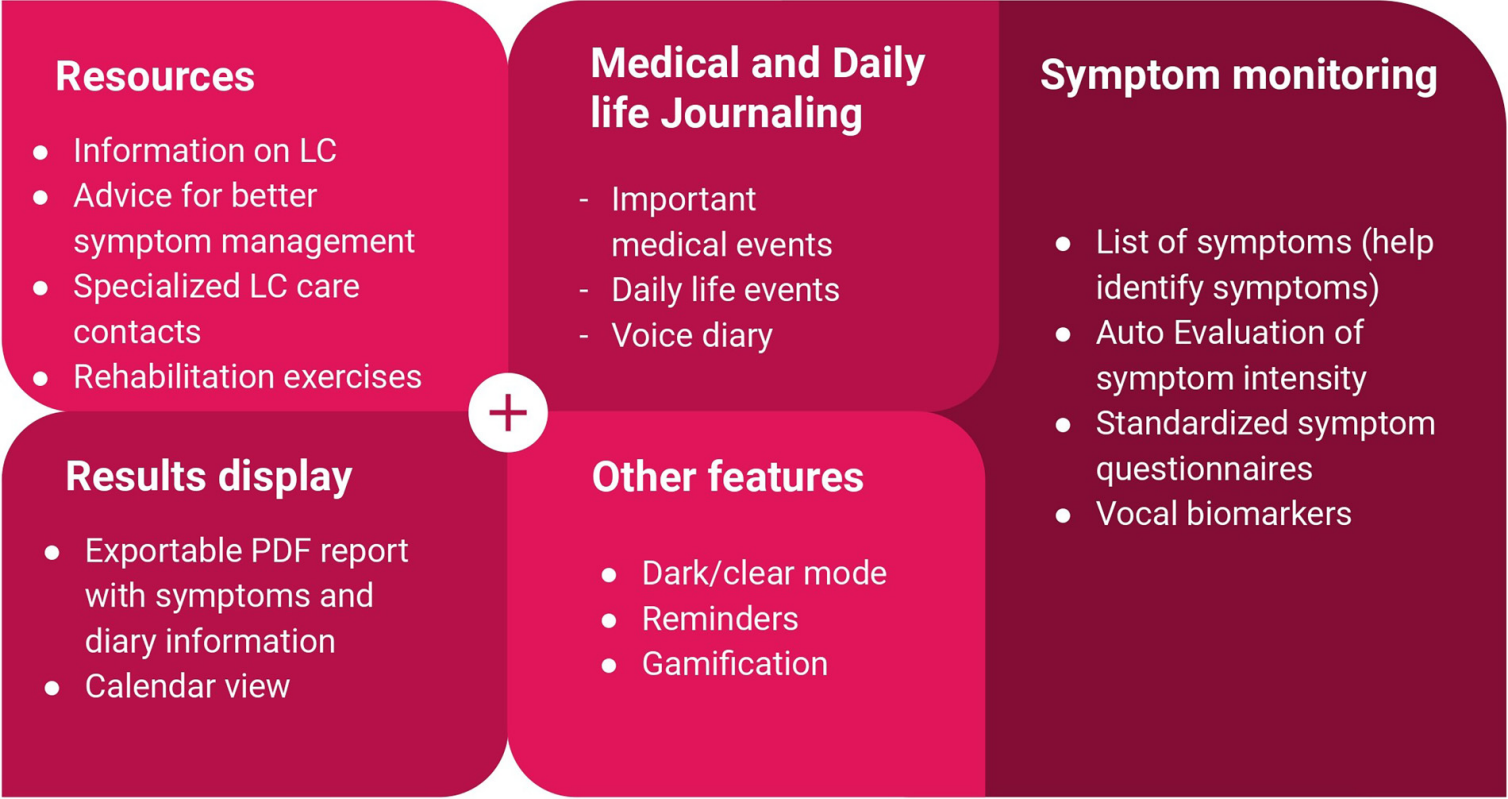
Main app functionalities.

The main functionalities expected to be present in the app are summarized in Figure 5.

### App development

Based on the app features defined above, a first version of a web-application has been developed with the help of patients at each stage to ensure that patients’ priorities, needs and the specificity of the disease were met. The web app format was chosen so that the app will be accessible using a smartphone or a computer.

The app was designed to be user-friendly, with a clear and guided manner to fill in the daily symptoms and health status. Briefly, after account creation, the users arrive at a home page with a message inviting them to complete their daily data.

Daily data include three steps: auto-evaluation of global health on a 0–10 Likert scale, auto-evaluation of symptoms chosen in an extensive list with their perceived intensity on a 0–5 scale, and finally a vocal diary allowing users to record the main information and feelings they experience.

Validated questionnaires for some specific symptoms or quality of life are accessible on the home page, as well as resources and the possibility to fill in a medical and daily life diary.

As vocal biomarkers are too early in their development and not validated yet, we decided to integrate them in a module with the option to do four voice recordings for research purposes, available on the home page.

A calendar view allows users to have an overview of their global health status using smileys, and to easily retrieve the days with medical or daily life events recorded.

Users also have the possibility to visualize the evolution of each symptom they recorded and of the questionnaire score in a simple and graphical manner, with the option to see the medical and daily life events so that a correlation could be done.

PDF reports summarizing data from a chosen period of time can be generated and downloaded with all these results to serve as a basis of discussion between PWLC and HCP.

**Figure 6.**
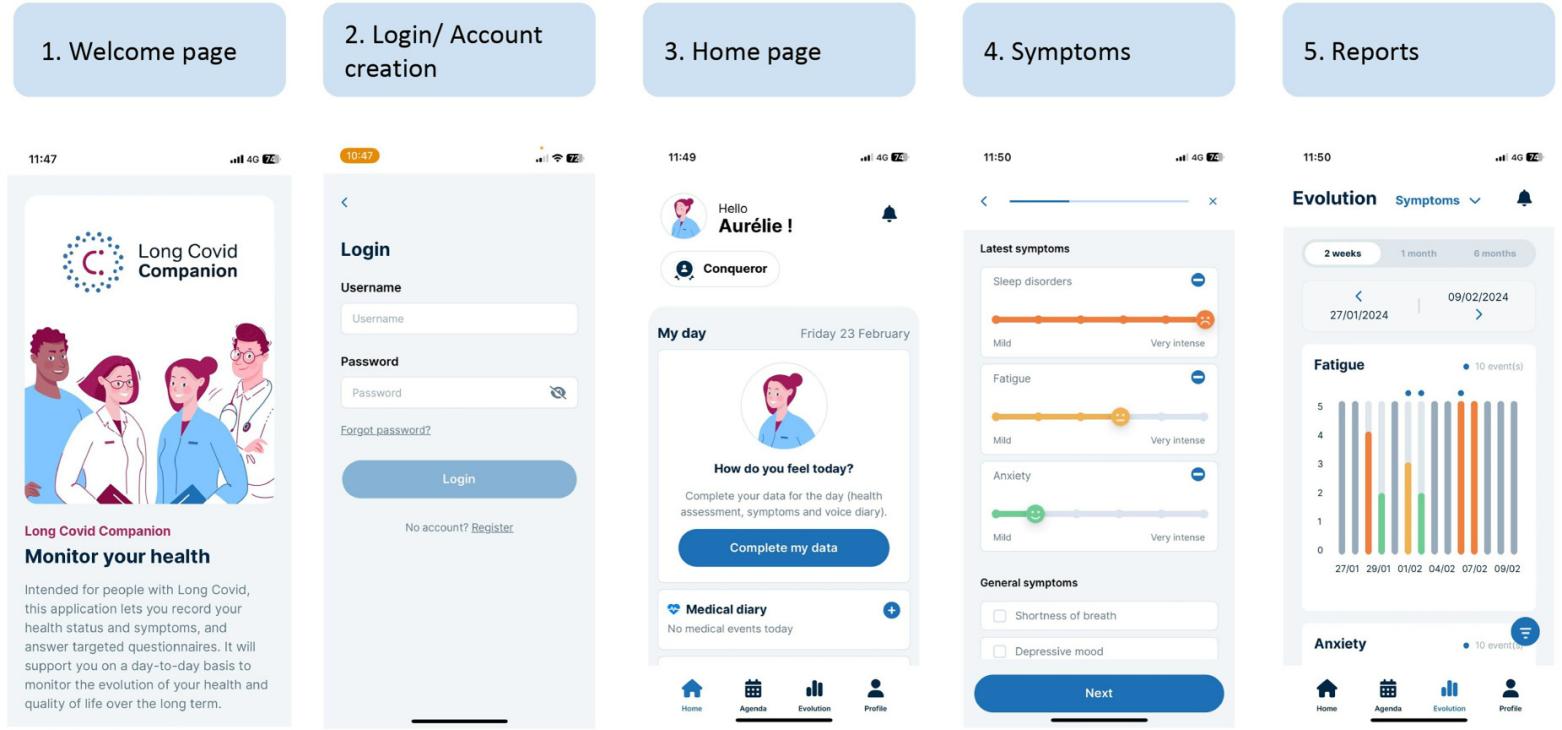
App user journey.

The overall app user journey is presented in Figure 6.

App contents, i.e., the list of symptoms, the validated questionnaires and the resources to integrate in the app, have been validated with our patient partners from the #apresJ20 Covid Long France Association who also tested a beta version of the app to identify and correct potential technical issues before organizing the focus group.

The app was presented during a focus group to six PWLC and three HCP. After a demonstration of the app, each main functionality was discussed one by one before opening the discussion. Feedback and improvement suggestions are summarized in Table 2.

**Table 2.** Focus group feedbacks.

Themes		Feedback (problems/suggestions)
Engagement	Reminders	“It would be nice, if possible, for us as patients to be able to choose whether or not to receive notifications.” “Studies that have been published on apps for therapeutic compliance, in which there are regular reminders, have shown that they can improve compliance.” “I completely agree that the intrusive aspect should be avoided” “I believe that a minimum recall frequency will make it useful.” “The goal would be to make it really customizable.” “Once a week is more reasonable (than every day).”
	Gamification	“Useful for some people, but not necessarily indispensable for everyone. It really depends on the person.”
Functionality	Completion daily information	“The fact that you can actually go into one place and have successive screens, that's very good. I really like it. Yes, I like it a lot. It's very clear. We go straight to the point.” “Likewise, I think it's clear. What's more, you move from the global to the more specific.”
	Self-evaluation global health perception	“It's good to have a tool that lets you quickly assess your health and how you feel.” “Being able to evaluate yourself like this, you can see that over the last month, there were times when things weren’t going so well, but overall, it wasn’t too bad.”
	Self-evaluation symptom intensity	“You can assess a lot of symptoms. It's pretty cool.” “Just be careful between the terminology of symptoms and diagnosis. Depression is a diagnosis, I don’t know what word could be used instead, but depressed mood, for example.” “It allows us to see the whole picture of our symptoms.” “It would be great to differenciate physical and mental health symptoms” “For the symptom list, did you used an official list from WHO or something else?”
	Standard questionnaires with scoring	“Indicate the scientific name of questionnaires on PDF reports, so that physicians can more likely know it” Only questionnaire scores will be available on reports, would it be interesting for HCP to have the details of responses to all questions? “The aim of the score is to have a sufficient value at the end.” “The doctor doesn’t necessarily have to look at the details of all the questions”
	Vocal diary	“I think that for the patients, it's something that's both very interesting and perhaps essential for them. Creating a narrative of their evolution in their own words, a kind of diary that also has a personal value.” “I find it interesting, but I don’t necessarily see what it can bring me” “It's a cathartic function for the patient, and in a concrete way, it helps reduce the deleterious effects of these complicated pathologies.” “It's like a personal diary, but vocal.”
	Calendar	“That's good. It gives a really global view over a whole month.” “it's very concise, very visual. And with the color codes, you can actually see the days when you’re better, when you’re worse, and that really stands out.” “it also lets you know whether I’ve been more or less involved in using the application, and whether I’ve invested more in it at one time or another.” It's good to have the option to add medical appointments in the future.
	Graphical report for symptoms and questionnaires	“It's very visual too, the colors, the evolution, you can follow the evolution over time very easily.” “It's a great benefit, it allows us to follow our progress over the long term.”
	Research module with voice recordings	“That's the heart of your project, I think, voice biomarkers.” “I’m rather curious to see what it will be like” “I’m all in favor” “Will we get feedback on the results of the recordings?” All participants would be willing to use this function.
	Daily life and medical diary	“It's quite important for pacing to be able to note on a daily basis the duration of our various activities and not only to mention ‘Intense’ ‘Moderate’ or ‘Mild.’” “it's always possible to use the ‘other’ fields or the voice diary” “there are days when I’m a little more willing. This can be recorded in the life diary too, in the other field, or in the voice diary.”
Information	Ressources	“The way resources are displayed as vignettes and links, it's very clear and visual.” “What's interesting is that you use the word encouragement and motivation, not reward.” “We shouldn’t feel bad if we don’t use the application every day and fill in our diary every day.”
	Chatbot integration	“Very good too, it's coherent in fact, it's a follow-up tool for the Long Covid, at the same time we also get instant answers to any questions we might have.” “You just have to make sure that the answers remain up to date with the latest data.”
Aesthetic	App colors	“Dark and clear mode is ergonomic”
Subjective items		“Well done, very well done. Honestly, there are a lot of points, the essential points of what we experience.” “Will the data be use for research purposes? “It is important to be informed on how and where our data will be stored” Does the app meet the needs? “Yes, a big yes” “There might also have been one point I’d have liked to see a little more, and that's the link with healthcare professionals.” “If we are too focused on our monitoring, it can also have, I think, a negative impact.” “Thank you very much for giving us a voice, for listening to us” “We’re trying to provide solutions to a real pathology. It's not the app that's going to get you hooked on your condition.”

The app received very good overall feedback from both PWLC and HCP: The app seemed to meet the needs of PWLC in many aspects and some of them expressed notable gratitude for the provision of this new tool. Participants appreciated the daily data completion journey, in particular to go from the more general (global health status), to the more specific (symptoms and vocal diary).

The symptom list was validated by the participants. One participant noticed that one item in the list was more a diagnosis than a symptom and recommended replacing “Depression” by “Depressed mood.”

The possibility to complete specific symptoms and quality of life questionnaires were mandatory for all participants. Some participants wondered if the questionnaires’ scientific name and the detailed responses to each item would be present on the PDF report. Whilst the scientific name was present (e.g., fatigue evaluation was labeled FSS9), only the overall score was integrated in the report because inclusion of detailed responses would have overloaded the reports. Two HCP present in the focus group ensured that the total score would be sufficiently informative to have a good overview.

One participant wondered whether there was a risk that the app would prompt users to stay focused on their disease and symptoms. A HCP present in the meeting answered that “We’re trying to provide a solution to a real pathology. It's not the app that's going to get you hooked on your condition.”

Some improvements suggested during the focus group were to provide additional ways to monitor mental health, to add or rename some symptoms, and to be able to record physical, intellectual, or meal activity in greater detail.

When presenting the research module with standardized voice recording for vocal biomarkers, participants confirmed that they were in favor of it and willing to have and use it. However, some participants were a bit disappointed that vocal biomarkers to measure some symptoms were not integrated in the app.

## Discussion

This study showed the entire co-design process of the Long COVID Companion app, designed with PWLC and HCP. The use of mixed methods allowed us (1) to obtain an in-depth comprehension of the daily problems of PWLC, the needs and expectations related to a voice-based monitoring tool, (2) to define the main characteristics the app, and (3) to validate a first version of the Long COVID Companion app.

The main needs and expectations identified in our study were to provide daily support on both daily life (psychological and administrative support) and medical aspects (help symptom identification and monitoring, improve communication between PWLC and HCP, limit medical visits, etc.).

The use of voice was generally well perceived and the expected added-value of using vocal biomarkers was to limit the fatigue of completing questionnaires or typing symptom description and to objectivize symptoms in comparison to auto-evaluation.

Barriers and fears for the use of a voice-based symptom monitoring app were principally linked to data privacy and the reliability of this new technology. This finding could be explained by the high level of education of participants and is in line with a previous study in the context of diabetes distress.^48^ This is an important aspect to be taken into account when developing voice-based technologies as voice is considered as sensitive and identifying data. Voice collection and analysis falls therefore under different regulations or laws, in particular GDPR in Europe or PIPEDA in Canada. In the United States, several laws exist at federal or state level. Finally, these different regulations do not protect individuals at the same level so it is highly recommended to obtain explicit informed consent for the users before voice collection.^49^

Based on our results, an ideal voice-based symptom monitoring app for PWLC should include the following items: (1) self-monitoring of symptoms, (2) standardized questionnaires for frequent symptoms or for quality of life assessment, (3) visualization and individual reports, (4) journaling, and (5) provide informative resources.

Regarding the self-monitoring module, it was critical to PWLC and HCP to provide a list of the main Long COVID-related symptoms to facilitate easier selection of those relevant to future users’ experience. In addition, we added the option to auto-evaluate perceived global health on a 0–10 Likert scale, which was meaningful for PWLC as it allowed them to evaluate their health at a global level and not focus only on individual symptoms. The use of vocal biomarkers for symptom monitoring was perceived as a promising approach, the advantages being the ease of use and the possibility to have a more objective evaluation than the auto-evaluation alone. There is, however, still a need to validate vocal biomarkers and to provide proof of their reliability.

The use of standardized questionnaires was an important feature to add as it was seen as a complement tool to assess symptoms and quality of life. The scientifically validated nature of the questionnaires was reassuring for PWLC. Moreover, obtaining scores on these questionnaires was perceived as tangible support facilitating discussion between PWLC and HCP. Despite standardized questionnaires being subject to bias (recall bias, respondent conscious or subconscious reaction, etc.)^50^ they remain perceived by PWLC as more objective than self-evaluation.

The graphical representation of symptom intensity and questionnaire score evolution as shown in Figure 5 was essential for both PWLC and HCP. This should help PWLC to interpret their data generated in the app and obtain a more objective overview of their health status in the past days or weeks. This function has already been shown to have an impact on engagement and motivation to use such a technology in the long term.^51^ HCP also found that the option to generate a PDF report with this graphical representation could be of interest during consultations, by summarizing their patient's symptom trajectories at a glance.

The journaling was very important for PWLC because of the fluctuant character of their disease and because many of them have cognitive impairments. The diary would recall what happened in the past and have reminders for upcoming medical appointments. The possibility to indicate the important medical and life events in parallel to the symptom monitoring could allow them to identify potential correlations between an event and symptom relapse or improvement. We also choose to integrate a vocal diary which will allow the users to easily record all their concerns and feelings. Previous studies suggested that written journaling could have a positive impact on mental health;^52^ however, there is a lack of consolidated results and long-term assessment of the benefit of using a diary. In other settings like postoperative period or patients with neuroendocrine tumors, it has been described that the use of a diary may reduce the recall biases and provide a broader picture of patients’ quality of life than traditional questionnaires.^53,54^

Voice journaling may have an additional value compared to written journaling by facilitating the diary completion and ultimately increasing the adherence to journaling.

Finally, different types of resources should be provided, in particular scientifically validated information on Long COVID. The aim of this module, however, would not only be to inform but also to help the app users to manage their disease. For example, tailored advice or rehabilitation exercises according to recorded symptoms could allow the app users to manage these symptoms. Information on specialized Long COVID consultation and administrative content on return to work and long-term disease recognition procedures could also be valuable.

The transferability of our results was also considered, defined as the extent to which it can be applied in other contexts or settings.^55^ The essential app functionalities identified in this study correspond to the three steps of the self-care process described by Grosjean et al. in the development of the e-CARE-PD study: monitoring, interpretation, and action.^51^ Some of the identified needs are specific to PWLC, such as obtaining help in symptom identification, but other concerns are common to people affected by other chronic diseases. In particular, among the most impacting symptoms, fatigue and anxiety were common to the vast majority of our PWLC study participants. These are common problems encountered in other chronic diseases like diabetes, migraine, or cancer.^56,57^ The use of voice and of vocal biomarkers was well accepted, the main identified advantages being to reduce fatigue due to the completion of questionnaires, to limit travel for medical appointments and to give an objective assessment of a symptom. As fatigue and mental health issues are central problems in many other chronic conditions these findings could be transferable to other diseases.^57–59^

Finally, we co-developed the first version of Long COVID Companion with the objective to release it as soon as possible to offer a supportive tool for PWLC who are in need of helpful, concrete solutions. The app was released as a companion tool and not yet as a medical device. Further functionalities could be added later, such as a module of rehabilitation exercises. Further developments will enable the certification of the app as a medical device and to negotiate the app reimbursement. Although the use of voice and vocal biomarkers was expected by many participants, we could not integrate vocal biomarkers in this first version as our vocal biomarker candidates require more validation before implementation. A direct interaction between PWLC and HCP was expected by some of the PWLC participants but could not be developed easily and rapidly as there were considerable feasibility issues to tackle, for example, the willingness of HCP to be involved in such a tool and the medical device regulations that would apply.^60^ This will be explored in future development of the Long COVID Companion app.

The study had several strengths. First, it was based on mixed methods, combining quantitative and qualitative data, providing a more in-depth comprehension of the unmet needs and the potential solutions to them. Second, we involved PWLC and HCP in charge of Long COVID patients as study participants, which is highly recommended when co-designing digital health solutions.^61^ In addition, we also involved PWLC and HCP as co-researchers throughout the entire study course, from the study conception to the study realization and valorization. They contributed to a better and complementary understanding of patient's needs and expectations that emerged from study results. The involvement of patients as co-researcher underlined the importance of checking for post-exertional malaise and raising the awareness of pacing to learn how to manage day-to-day activities. They also proposed to integrate directly in the app their chatbot on Long COVID previously developed with PWLC from ApresJ20 Covid Long France and HCP in order to facilitate tailored answers to patients’ needs. Our study participants, in both PWLC and HCP categories, had a wide age and education level range, showing that older people or those with lower levels of education, usually under-diagnosed, were interested in and willing to use such a voice-based monitoring app. Long Covid Companion app is, to our knowledge, the first app intended for PWLC entirely co-designed with them that will be available in three languages and free of charge in Europe. Similar co-design of digital health intervention studies exist in other therapeutic areas like Parkinson's disease, type 2 diabetes, or endometriosis;^29,51,62^ however, our study is to our knowledge the only one intended for PWLC.

The study also had some limitations. There was an overrepresentation of women and of highly educated people in the survey and in the interviews. This is not surprising, as women are more frequently affected by Long COVID and more willing to participate in studies.^63^ A majority of PWLC participating in the survey were already diagnosed, had severe Long COVID symptoms and were in a specialized Long COVID care. Therefore, the interest in a symptom monitoring app might be different, and the needs and expectations might not be generalizable to the entire population of Long COVID patients, particularly those who are not in specialized care and lack an official Long COVID diagnosis whose needs may differ to our study population. The study was conducted only in French, so the app evaluation in the context of other languages and target populations will be necessary. We used ad-hoc questionnaires for the survey and to guide the interviews and no validated ones, moreover self-reported questionnaires are prone to desirability, recall, and response bias. However, as questionnaires were reviewed by PWLC and HCP before the study started, this mitigated the risk and allowed to obtain a comprehensive understanding of the problems and needs of PWLC and the risk is limited to impact app use and benefit. Finally, there are also some limitations regarding vocal biomarkers that need clinical validation before their use in daily practice and with most of them still at a research stage.

## Conclusion

We co-developed, with patients and HCPs, a voice-based app to monitor Long COVID symptoms based on the expectations and needs of PWLC. This digital health solution addresses a major gap and provides day-to-day support for people affected by Long Covid. We have also demonstrated that they have high expectations for the use of vocal biomarkers. Future longitudinal studies will be necessary to evaluate acceptability, usability, and effectiveness of the Long COVID Companion app for Long COVID care and disease management, but also to validate the vocal biomarkers candidate of Long COVID symptoms. Future app certification as a medical device will also allow to integrate it in the healthcare pathway of PWLC.

## Supplemental Material

sj-docx-1-dhj-10.1177_20552076241272671 - Supplemental material for Co-design of a voice-based app to monitor long COVID symptoms with its end-users: A mixed-method study Supplemental material, sj-docx-1-dhj-10.1177_20552076241272671 for Co-design of a voice-based app to monitor long COVID symptoms with its end-users: A mixed-method study by Aurélie Fischer, Gloria Aguayo, India Pinker, Pauline Oustric, Tom Lachaise, Paul Wilmes, Jérôme Larché, Charles Benoy and Guy Fagherazzi in DIGITAL HEALTH

sj-xlsx-2-dhj-10.1177_20552076241272671 - Supplemental material for Co-design of a voice-based app to monitor long COVID symptoms with its end-users: A mixed-method study Supplemental material, sj-xlsx-2-dhj-10.1177_20552076241272671 for Co-design of a voice-based app to monitor long COVID symptoms with its end-users: A mixed-method study by Aurélie Fischer, Gloria Aguayo, India Pinker, Pauline Oustric, Tom Lachaise, Paul Wilmes, Jérôme Larché, Charles Benoy and Guy Fagherazzi in DIGITAL HEALTH

sj-xlsx-3-dhj-10.1177_20552076241272671 - Supplemental material for Co-design of a voice-based app to monitor long COVID symptoms with its end-users: A mixed-method study Supplemental material, sj-xlsx-3-dhj-10.1177_20552076241272671 for Co-design of a voice-based app to monitor long COVID symptoms with its end-users: A mixed-method study by Aurélie Fischer, Gloria Aguayo, India Pinker, Pauline Oustric, Tom Lachaise, Paul Wilmes, Jérôme Larché, Charles Benoy and Guy Fagherazzi in DIGITAL HEALTH

sj-xlsx-4-dhj-10.1177_20552076241272671 - Supplemental material for Co-design of a voice-based app to monitor long COVID symptoms with its end-users: A mixed-method study Supplemental material, sj-xlsx-4-dhj-10.1177_20552076241272671 for Co-design of a voice-based app to monitor long COVID symptoms with its end-users: A mixed-method study by Aurélie Fischer, Gloria Aguayo, India Pinker, Pauline Oustric, Tom Lachaise, Paul Wilmes, Jérôme Larché, Charles Benoy and Guy Fagherazzi in DIGITAL HEALTH

sj-xlsx-5-dhj-10.1177_20552076241272671 - Supplemental material for Co-design of a voice-based app to monitor long COVID symptoms with its end-users: A mixed-method study Supplemental material, sj-xlsx-5-dhj-10.1177_20552076241272671 for Co-design of a voice-based app to monitor long COVID symptoms with its end-users: A mixed-method study by Aurélie Fischer, Gloria Aguayo, India Pinker, Pauline Oustric, Tom Lachaise, Paul Wilmes, Jérôme Larché, Charles Benoy and Guy Fagherazzi in DIGITAL HEALTH
