# Supplementary material for: Co-design of a voice-based app to monitor long COVID symptoms with its end-users: A mixed-method study
Source: Digit Health. 2024 Sep 9;10:20552076241272671. doi: 10.1177/20552076241272671 (PMC11384972; doi:10.1177/20552076241272671)
Supplement: sj-docx-1-dhj-10.1177_20552076241272671 - Supplemental material for Co-design of a voice-based app to monitor long COVID symptoms with its end-users: A mixed-method study [file sj-docx-1-dhj-10.1177_20552076241272671.docx]

# Supplementary file 1

# Semi-structured interview guide

1/ Introduction

Introduce the interviewer, the study objectives and the interview procedure.

Remind participants that they have given their consent to participate, and that the interview will be audio recorded. Check that the participant still agrees.

2/ Interview

Collect socio-demographic data:

- participant category: PWLC or HCP
- age
- education level

Following questions are used to guide the interview but are not exhaustive and are adaptable to the participant’s answers and reactions.

Impact of Long COVID

- What are your (your patients) most impacting symptoms?
- What difficulties do you (your patients) encounter in the daily life
- Would you be interested in a symptom monitoring app for you or your patients, and why? (do you already track your symptoms? In which manner?)
- How could such an app help you/your patients on a daily basis?
- What advantages do you see in using a symptom monitoring application?
- Did Long COVID have an impact on return to work or leisure activities (for you/your patients)? Could the app provide any help in this area?

Voice use

- Imagine that you (or your patients) use an app to monitor symptoms. How do you think voice could be used in this way?
- Do you think it's possible to measure symptoms in the voice using voice biomarkers? What advantages do you see?
- Do you have any fears about such a voice-based application? If so, what are they? What do you see as the drawbacks or barriers?

App characteristics

- What frequency of use do you consider acceptable for a symptom monitoring app? for voice recordings? for questionnaires?
- What should be included in addition to symptom monitoring?
- What do you think of the possibility of integrating a module offering rehabilitation exercises? How do you see it?
- What do you think should be implemented in the application if a worsening of symptoms is detected?
- What do you think about integrating personalized advice into the application? Is it important to you?
- Should the application include information on COVID Long? What do you think?
- How do you imagine the application in terms of interactions with other people and the sharing of results?
- What features could help you/your patients use the application over the long term?
- Would you be willing to make several successive voice recordings? If so, what kind of recordings (standardized, free, etc..)

# Focus group guide

1/ Introduction

Presentation of the focus group moderator, reminder of study aims and status.

2/ Long COVID App demo

3/ Discussion of different app functionalities

- Self-assessment of overall health
- Self-assessment of symptom intensity
- User journey for daily health assessment
- In-depth symptom questionnaires with scores
- Audio recordings for research
- Voice diary
- Calendar view
- Graphical display of results
- Life journal
- Resources displayed on home page
- Integration of chatbot

4/ Open discussion - feedbacks

Stimulating questions:

- What is your first impression of the application?
- Does the application correspond to the idea you had of it? Why or why not?
- Do you think this application meets the expectations of people affected by Long Covid?
- Do you see any negative points or potential barriers to using the app?
- What do you think of the content we integrated into the prototype?
